# Sex and race differences in J-Tend, J-Tpeak, and Tpeak-Tend intervals

**DOI:** 10.1038/s41598-019-56328-8

**Published:** 2019-12-27

**Authors:** Katerina Hnatkova, Ondřej Toman, Martina Šišáková, Peter Smetana, Katharina M. Huster, Petra Barthel, Tomáš Novotný, Georg Schmidt, Marek Malik

**Affiliations:** 1 National Heart and Lung Institute, Imperial College, 72 Du Cane Road, Shepherd’s Bush, London, W12 0NN England; 2 Department of Internal Medicine and Cardiology, University Hospital Brno, Faculty of Medicine, Masaryk University, Jihlavská 20, 625 00 Brno, Czech Republic; 3 Wilhelminenspital der Stadt Wien, Montleartstraße 37, 1160 Vienna, Austria; 4 Klinikum rechts der Isar, Technische Universität München, Ismaninger Straße 22, D-81675 Munich, Germany

**Keywords:** Cardiovascular biology, Medical research

## Abstract

To facilitate the precision of clinical electrocardiographic studies of J-to-Tpeak (JTp) and Tpeak-to-Tend (Tpe) intervals, the study investigated their differences between healthy females and males, and between subjects of African and Caucasian origin. In 523 healthy subjects (254 females; 236 subjects of African origin), repeated Holter recordings were used to measure QT, JT, JTp, and Tpe intervals preceded by both stable and variable heart rates. Subject-specific curvilinear regression models were used to obtain individual QTc, JTc, JTpc and Tpec intervals. Rate hysteresis, i.e., the speed with which the intervals adapted after heart rate changes, was also investigated. In all sex-race groups, Tpe intervals were not systematically heart rate dependent. Similar to QTc intervals, women had JTc, and JTpc intervals longer than males (difference 20–30 ms, p < 0.001). However, women had Tpec intervals (and rate uncorrected Tpe intervals) shorter by approximately 10 ms compared to males (p < 0.001). Subjects of African origin had significantly shorter QTc intervals than Caucasians (p < 0.001). Gradually diminishing race-difference was found for JTc, JTpc and Tpec intervals. JTc and JTpc were moderately increasing with age but Tpe/Tpec were not. Rate hysteresis of JTp was approximately 10% longer compared to that of JT (p < 0.001). In future clinical studies, Tpe interval should not be systematically corrected for heart rate and similar to the QT interval, the differences in JT, JTp and Tpe intervals should be corrected for sex. The differences in QT and JT, and JTp intervals should also be corrected for race.

## Introduction

During the past decade, number of published studies were devoted to the association of cardiovascular and arrhythmic risk with changes of sub-sections of the QT interval measured on standard electrocardiograms (ECG)^1^. Whilst some studies reported that the prolongation of the interval between the peak and the offset of the T wave (the Tpe interval) is indicative of increased risk^2^, other studies disagreed and related increased risk to the prolongation of the interval between the J point and the peak of the T wave (the JTp interval)^3^. Changes of the JTp interval rather than that of Tpe have also been reported to distinguish between QT interval prolonging drugs that are pure blockers of the delayed potassium rectifier channel and those influencing multiple ion channels involved in myocardial repolarisation^4,5^.

Compared to the measurement of ECG intervals, more advanced ECG processing techniques likely offer more accurate estimates of the distribution of repolarisation timing and synchrony across ventricular myocardium^6,7^. Nevertheless, monitoring and comparing the ECG intervals is clearly more practical and perhaps somewhat less dependent on the quality of ECG recordings. Further studies are therefore still needed to solve the present conundrum of the electrophysiologic properties and meaning of the ECG intervals involving the T wave peak.

Among others, it is not known whether future risk prediction studies based on the JTp and Tpe intervals should use different sex-specific limits of normality comparable to the well-known sex-specific QTc interval dichotomies^8^. Similarly, race influence on the normal values of the JTp and Tpe intervals has been little studied.

To contribute to this knowledge gap, we have investigated the position of the T peak in ECGs extracted from 12-lead Holter recordings of a relatively large population of healthy volunteers of both sexes involving approximately the same numbers of subjects of African and Caucasian origin. In each subject, the Holter recordings included sections of substantially different heart rates which allowed us studying individual heart rate profiles, thus eliminating the problem of inaccurate heart rate correction.

Whilst the main goal of the study was to investigate the sex and race differences in the JT, JTp and Tpe intervals, the accuracy of the data that we report depends on the validity of rate corrections. Therefore, we include justification of the rate correction technology^9^ and show that the accuracy of the correction is sex and race independent.

## Methods

### Investigated population

Two pharmacology studies were conducted in healthy subjects and organised at specialised clinical research laboratories. All subjects participating at the studies had normal screening ECG and normal clinical investigation^10^. In each subject, demographic data were collected, and self-declared race classification was obtained. Body mass index (BMI) was calculated as the body weight in kilograms divided by squared body height in meters.

The original studies were approved by the relevant ethics boards (Parexel International, Baltimore; California Clinical Trials, Glendale; Spaulding Clinical Services, Milwaukee) and all participants gave written informed consent in accordance with the Helsinki declaration. Since only baseline off-treatment data were analysed in the present investigation, other details of the source studies are irrelevant. For the same reason as well as because reported data were available only with pseudo-anonymised identifiers, no separate ethics clearance of the present investigation was required as per the local legislation.

### Investigative protocol

In each participant, repeated 12-lead day-time Holter recordings were made during multiple baseline days when the subjects were on no treatment. During these off-treatment days, study participants performed repeated postural provocative manoeuvres for the purposes of capturing wide ranges of heart rates on the Holters^11^. The manoeuvres included position changes between supine, unsupported sitting, and unsupported standing positions, each maintained for a minimum of 10 minutes with the changes between the positions completed, per protocol, within less than 20 seconds. The Holter recordings used Mason-Likar electrode positions.

### Electrocardiographic measurements

Using previously published methods^11^, QRS onset, QRS offset (i.e., the J point), and T wave offset were measured in multiple samples taken from the 12-lead Holter recordings. The measurements were made in representative morphologies derived from 10-second ECG segments and were sampled at 1000 Hz. Pattern matching algorithms^12^ were also used to ensure that similar morphologies of the QRS onset and offset and of T wave offset were measured similarly. Quality control of the measurements included visual verification and manual correction of computerized measurements by at least two independently working cardiologists with data reconciliation in cases of their disagreement. The reconciliation of the differences between independently working observers and the use of pattern matching adjustment algorithms eliminated the problem of intra- and inter-observer variability^13^.

For each measured ECG sample, a 5-minute history of RR interval preceding the measurements was also obtained. The measurements were made in ECG segments that were preceded by both stable and variable heart rate during the preceding 5 minutes.

**Figure 1:**
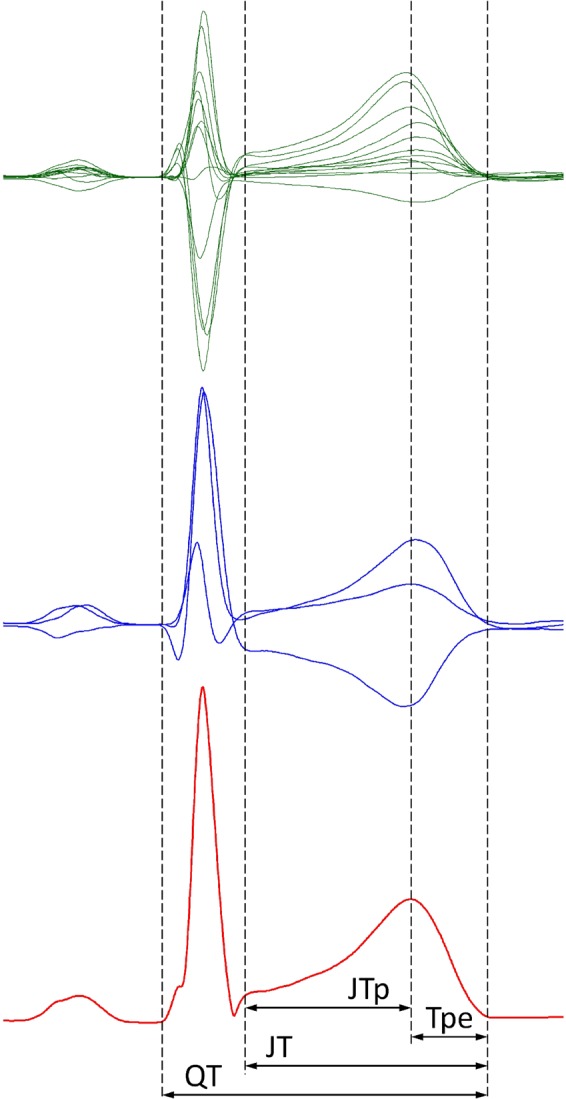
Schema of the ECG interval measurement. The top green panel shows the representative beatforms of all 12 ECG leads superimposed on the same isoelectric axis. The middle blue panel shows the derived orthogonal XYZ leads. The bottom red panel shows the vector magnitude of the orthogonal XYZ leads. The vertical dashed lines show the positions of QRS onset, QRS offset (J point), T wave peak, and T wave offset. The interval measurements are shown below the bottom panel. Note that while the 3 panels are synchronised along the horizontal time axis, the scales of the vertical axes are different for the panels. Note also in the top panel that the position of the T wave peak is slightly lead dependent.

Using a published transformation matrix^14^, orthogonal XYZ leads were derived from each representative morphology of a 10-second ECG segment with measured QRS complex and T wave offset. Vector magnitude of the XYZ leads was constructed and previously validated algorithm^15^ was used to detect the T wave peak of this vector magnitude^16^. This divided the JT interval into the JTp interval (between the J point and the peak of the T wave) and the Tpe interval (between the peak and the offset of the T wave). Figure 1 shows a schema of the measurement.

### Heart rate and rate hysteresis correction

In each study participant, data from multiple Holter recordings were pooled together and, based on an earlier report^9,17^, the following curvilinear regression was used to investigate the subject-specific relationship between the QT intervals and the underlying heart rate:

$${{\rm{QT}}}_{i}=\alpha +\frac{\delta }{\gamma }({{\rm{RR}}}_{i}^{\gamma }-1)+{\varepsilon }_{i},$$

where QT_*i*_ are individual QT interval measurements, *RR*_*i*_ are RR intervals durations (all expressed in seconds) that represent the corresponding underlying heart rate, *α*, *δ*, and *γ* are the subject-specific central value, slope, and curvature of the QT/RR relationship, respectively, and *ε*_*i*_ are normally distributed zero centred regression errors (here, for each study subject separately, the indexes *i* represent multiple measurements of the QT interval duration and of its underlying heart rate – see the following paragraph on heart rate hysteresis).

This curvilinear regression leads to subject-specific heart rate correction in the form $QTc=QT+\frac{\delta }{\gamma }(1-R{R}^{\gamma })$. This was applied to all QT interval measurements in the given subject and the average of all rate corrected QT interval values was obtained. The symbol QTc will be further used to denote this subject-specific averaged rate corrected QT interval. (It is easy to see that in each subject, QTc equals to the central value *α* of the curvilinear regression).

Since QT interval duration does not depend on the preceding RR interval duration but on the underlying heart rate, correction for QT/RR hysteresis was incorporated using the exponential decay model^18^. That is, for a QT interval reading preceded by RR interval sequence ${\{R{R}_{i}\}}_{i=0}^{N}$ (*RR*_0_ closest to the QT measurement), coefficient $\Lambda (k)=\mathop{\sum }\limits_{i=0}^{k}R{R}_{i}$ was calculated, and the QT interval was related to the value $RR^{\prime} =\mathop{\sum }\limits_{i=0}^{N}{\omega }_{i}R{R}_{i}$, where for each $j=0,\cdots ,N$, $\mathop{\sum }\limits_{i=0}^{j}{\omega }_{i}=(1-{e}^{-\lambda \Lambda (j)/\Lambda (N)})/(1-{e}^{-\lambda })$. The coefficient *λ* characterized the subject-specific QT/RR hysteresis, i.e. the speed with which QT interval adapted to a change in the underlying heart rate. The coefficient *λ* was obtained by minimizing the regression residual of the curvilinear regression between the QT intervals and the RR intervals of the underlying rate (i.e., minimizing the standard deviation of the *ε*_*i*_ errors). The value of *λ* was subsequently converted into the so-called hysteresis time-constant, i.e. the time needed for the QT interval to reach 95% of its new value after a heart rate change.

The very same approach as for the QT intervals was used for the JT intervals and for the JTp intervals. That is, in each study subject, the exactly same regression modelling formulae as described for the QTc intervals were used to obtain the slopes, curvatures, and hysteresis time-constants for the JT/RR and JTp/RR relationships together with the subject-specific rate corrected JTc and JTpc intervals. The subject-specific rate corrected Tpe interval was obtained as the difference TPec = JTc − JTpc.

### Subject specific interval values

To characterize individual subjects of the study, the heart rate corrected QTc, JTc, JTpc, and Tpec intervals measured in all ECGs of a given study subject were averaged. The same was applied to rate uncorrected Tpe intervals (within-subject averages of rate uncorrected QT, JT, and JTp intervals were also obtained but are not presented).

### Justification of the heart rate and rate hysteresis correction

The incorporation of the rate hysteresis into the heart rate correction of ECG intervals has previously been validated^9,18–20^. Nevertheless, these previous studies have not considered combined sex and race differences. Therefore, we have also repeated the justification of the rate hysteresis correction in the subsets of the present study.

In addition to the curvilinear heart rate correction $$QTc=QT+\frac{\delta }{\gamma }(1-R{R}^{\gamma })$$, we also considered Framingham correction^21^ $$QTc=QT+0.154(1-RR)$$ and a subject-specific linear heart rate correction $$QTc=QT+$$$$\beta (1-RR)$$, where the coefficient *β* was optimized in each subject in order to achieve, in each subject separately, zero correlation between QTc and RR interval values (the interval durations are still expressed in seconds).

Both the Framingham and the linear corrections were applied to (a) RR intervals obtained as the average of the last 3 RR intervals preceding the QT interval measurement - that is, the last 3 RR intervals of the ECG segment in which the representative QRS-T morphology was obtained, and (b) RR intervals values obtained as the average of all the RR intervals within the 10-second ECG segment providing the representative QRS-T morphology. The linear correction was also applied to the RR interval value obtained by the means of universal hysteresis correction^20^ that assumed a hysteresis time-constant of 2 minutes.

Together with the curvilinear heart rate correction, this provided 6 different methods to obtain the QTc values. The performance of these methods was compared by calculating the intra-subject standard deviations of QTc intervals. A method was considered more accurate compared to a different method if it provided statistically significantly lower intra-subject standard deviation of QTc intervals across all sex- and race-defined sub-populations of the study.

In order to assess the practical implications of the differences between the correction possibilities, we have also investigated the 80% range of QTc values in each subject. That is, with each correction method and for each study subject, the difference between the 10^th^ and 90^th^ percentile of the QTc values was used as an indicator of the intra-subject variability of QTc measurements.

The very same approach of comparing different correction methods was used for heart rate correction of JT and JTp intervals.

### Statistics and data presentation

Continuous data are presented as mean ± standard deviation (SD). Comparison of continuous data between sexes and between race-specific groups were tested by two-sample two-tail t-test assuming different variances; within-subject comparisons of continuous data were tested by two-tail paired pair t-test. Dependency between variables was assessed by Pearson correlation coefficients and graphically displayed by linear regressions shown together with their 95% confidence intervals. Statistical tests were performed using the SPSS Statistics 64-bit version 25 package (IBM, Armonk, NY, USA). P values < 0.05 were considered statistically significant. Because of the interdependency of compared data, no adjustment for multiplicity of statistical testing was made. All statistical comparisons that were performed are described in the Result section.

## Results

### Population

Taken together, the two source pharmacological studies investigated 523 healthy subjects. Of these, 254 (48.6%) were females. Altogether, 236 (45.1%) and 259 (49.5%) subjects categorised themselves as of African and Caucasian origin, respectively. The other declared races included Asian, Polar region natives, and non-specified “Other race group”. Age of the subjects at the time of Holter recordings and their BMI are shown in Table 1; there were no significant differences between the ages or BMI of different sex- and race-specific subgroups.

**Table 1:** Study population and ECG data.

	Female			Male			P value	
	African	Caucasian	Other	African	Caucasian	Other	F vs M	A vs C
N=	111	130	13	125	129	15		
Age [years]	31.9 ± 8.9	34.6 ± 8.9	33.5 ± 11.2	34.2 ± 7.7	33.4 ± 7.8	32.1 ± 7.9	NS	NS
BMI [kg/m^2^]	25.9 ± 1.7	24.6 ± 1.6	25.9 ± 1.6	26.1 ± 1.8	24.2 ± 1.8	25.3 ± 1.8	NS	NS
ECG measurements	1265 ± 211	1221 ± 233	1276 ± 229	1249 ± 240	1278 ± 195	1369 ± 153	NS	NS
QT/RR hysteresis	112.3 ± 20.9	112.2 ± 19.9	106.1 ± 13.7	121.8 ± 24.0	118.6 ± 20.8	115.2 ± 18.4	< 0.001	NS
QT/RR curvature	0.600 ± 0.595	0.617 ± 0.694	0.806 ± 0.691	0.944 ± 0.830	0.699 ± 0.581	0.904 ± 0.486	0.001	0.048
QT/RR slope	0.161 ± 0.033	0.157 ± 0.028	0.171 ± 0.034	0.139 ± 0.024	0.141 ± 0.023	0.145 ± 0.024	< 0.001	NS
QTc [ms]	415.2 ± 13.0	421.1 ± 12.8	425.5 ± 11.7	394.6 ± 12.4	402.9 ± 10.8	396.4 ± 11.2	< 0.001	< 0.001
JT/RR hysteresis	116.4 ± 21.3	117.0 ± 19.7	111.8 ± 13.7	128.0 ± 27.0	125.3 ± 23.4	121.6 ± 18.9	< 0.001	NS
JT/RR curvature	0.623 ± 0.595	0.610 ± 0.672	0.798 ± 0.616	0.903 ± 0.794	0.675 ± 0.544	0.853 ± 0.443	0.004	0.033
JT/RR slope	0.161 ± 0.034	0.157 ± 0.029	0.174 ± 0.037	0.138 ± 0.024	0.140 ± 0.023	0.144 ± 0.024	< 0.001	NS
JTc [ms]	317.9 ± 13.4	321.5 ± 13.3	325.6 ± 11.7	293.0 ± 12.7	298.1 ± 11.6	292.1 ± 11.6	< 0.001	0.001
JTp/RR hysteresis	123.2 ± 32.9	135.0 ± 28.1	128.3 ± 26.3	138.2 ± 35.8	140.5 ± 30.4	130.8 ± 24.2	0.001	0.024
JTp/RR curvature	0.517 ± 0.846	0.377 ± 1.113	0.657 ± 0.7	0.851 ± 1.352	0.300 ± 0.673	0.652 ± 0.904	NS	< 0.001
JTp/RR slope	0.165 ± 0.050	0.149 ± 0.071	0.177 ± 0.049	0.142 ± 0.107	0.126 ± 0.023	0.135 ± 0.024	< 0.001	0.021
JTpc [ms]	230.9 ± 15.9	233 ± 15.1	239.4 ± 11.2	198.8 ± 15.7	203 ± 12.4	193.7 ± 15.0	< 0.001	0.033
Tpe [ms]	88.4 ± 7.2	87.7 ± 6.4	87.8 ± 6.6	94.2 ± 6.3	94.3 ± 5.7	97.5 ± 5.5	< 0.001	NS
Tpec [ms]	87.1 ± 7.7	88.5 ± 7.1	86.3 ± 5.6	94.2 ± 9.1	95.2 ± 6.0	98.4 ± 7.6	< 0.001	NS

For the different sub-groups of the study population, the table shows the number of study subjects (N=), their ages, the numbers of ECG measurements per subject, and the different ECG measurement values. For each of the ECG measurement values, the p-values are shown for statistical comparisons between females and males (F vs M) and between subjects of African and Caucasian origin (A vs C). Further details of the statistical comparisons between the groups are presented in the text. NS – not significant. The numerical values are means ± standard deviations.

In total, the study analysis was based on 657,134 ECG readings. There were no significant differences between the numbers of ECG readings per subject in different sex- and race-specific groups (Table 1).

### ECG intervals and closeness of fit of curvilinear regressions

The curvilinear regression models with rate hysteresis correction fitted the data closely. For QT, JT, and JTp intervals, the regression residuals (i.e. that intra-subject SD of QTc, JTc, JTpc values obtained with the subject-specific curvilinear correction formula) were only 5.60 ± 1.10 ms, 5.60 ± 1.16 ms, and 7.24 ± 2.19 ms, respectively.

**Figure 2:**
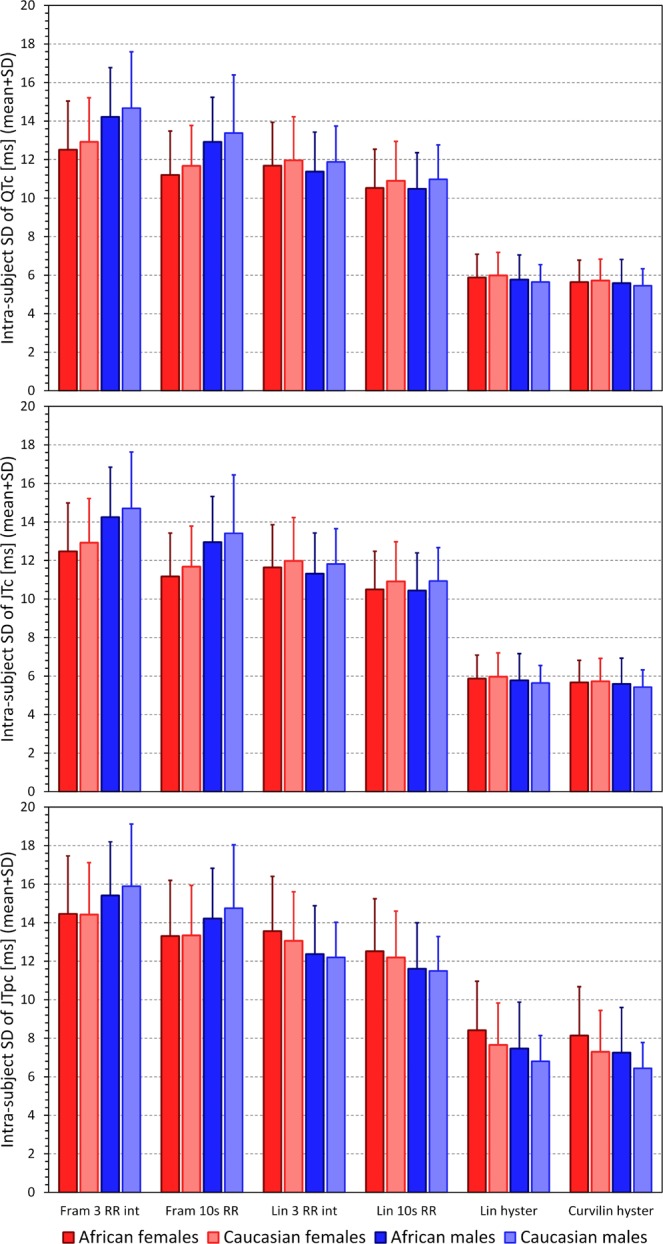
Summary of intra-subject standard deviations of QTc (top panel), JTc (middle panel) and JTpc (bottom panel) intervals obtained with Framingham formula using the average of last 3 RR interval preceding the interval measurement (Fram 3 RR int), Framingham formula using the 10-second average of RR intervals (Fram 10 s RR), subject-specific linear formula using the average of last 3 RR interval preceding the interval measurement (Lin 3 RR int), subject-specific linear formula using the 10-second average of RR intervals (Lin 10 s RR), subject-specific linear formula using universal rate hysteresis correction (Lin hyster), and curvilinear subject-specific formula using subject-specific hysteresis correction (Curvilin hyster). The population mean + standard deviations of the intra-subject standard deviations are shown for each of the sex- and race-defined (African vs Caucasian) sub-population. In each of these sub-populations, the intra-subject standard deviations of all the intervals obtained with curvilinear model + hysteresis correction were statistically significantly smaller compared to all other correction possibilities (p < 0.001 for all).

On the contrary, Fig. 2 shows that the omission of rate hysteresis led to substantial increases in the intra-subject SD of the rate corrected intervals. The subject-specific linear model involving the rate hysteresis led to intra-subject SD of QTc, JTc, and JTpc intervals only marginally higher than those obtained with the curvilinear model but the reduction of the intra-subject SD of the intervals corrected by the curvilinear model was still highly statistically significant, p < 0.001 for all sex- and race-defined (African vs Caucasian) sub-populations.

**Figure 3:**
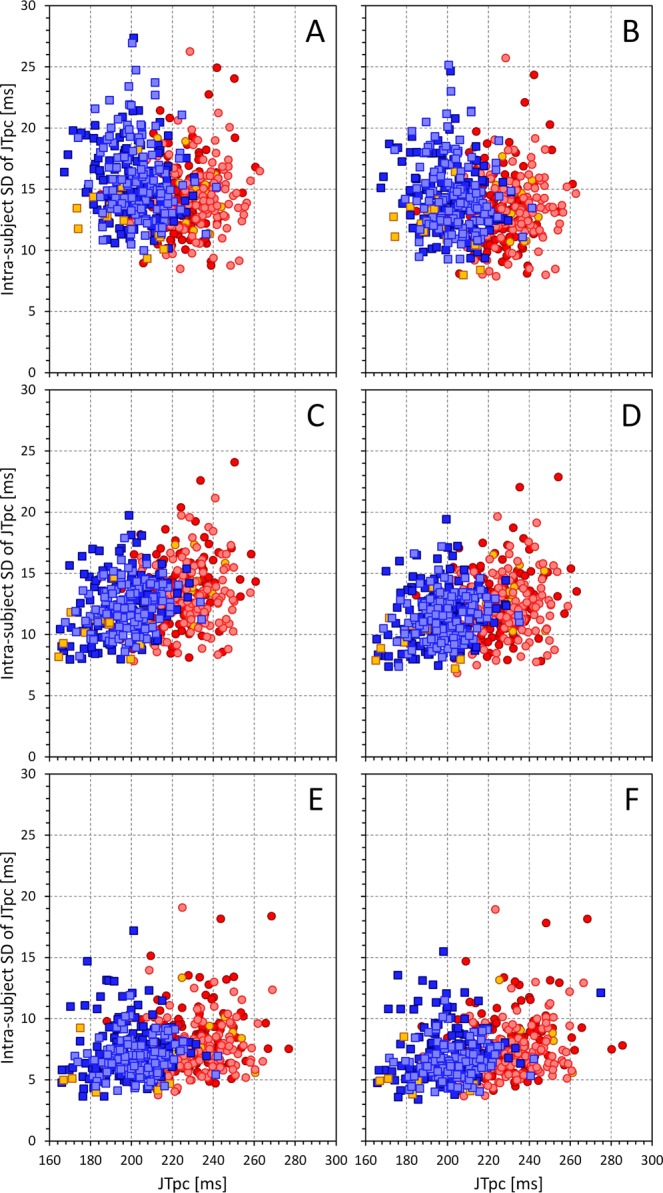
Graphical display of the gradual reduction of the intra-subject standard deviations of JTpc intervals (vertical axes) and of the changes of their intra-subject mean values (horizontal axes) by different rate corrections: Framingham formula using the average of last 3 RR interval preceding the interval measurement (panel A), Framingham formula using the 10-second average of RR intervals (panel B), subject-specific linear formula using the average of last 3 RR interval preceding the interval measurement (panel C), subject-specific linear formula using the 10-second average of RR intervals (panel D), subject-specific linear formula using universal rate hysteresis correction (panel E), and curvilinear subject-specific formula using subject-specific hysteresis correction (panel F). In each panel, the dark red circles, light red circles, amber circles, dark blue squares, light blue squares, and amber squares correspond to the measurements in African females, Caucasian females, other race females, African males, Caucasian males, and other race males, respectively.

**Figure 4:**
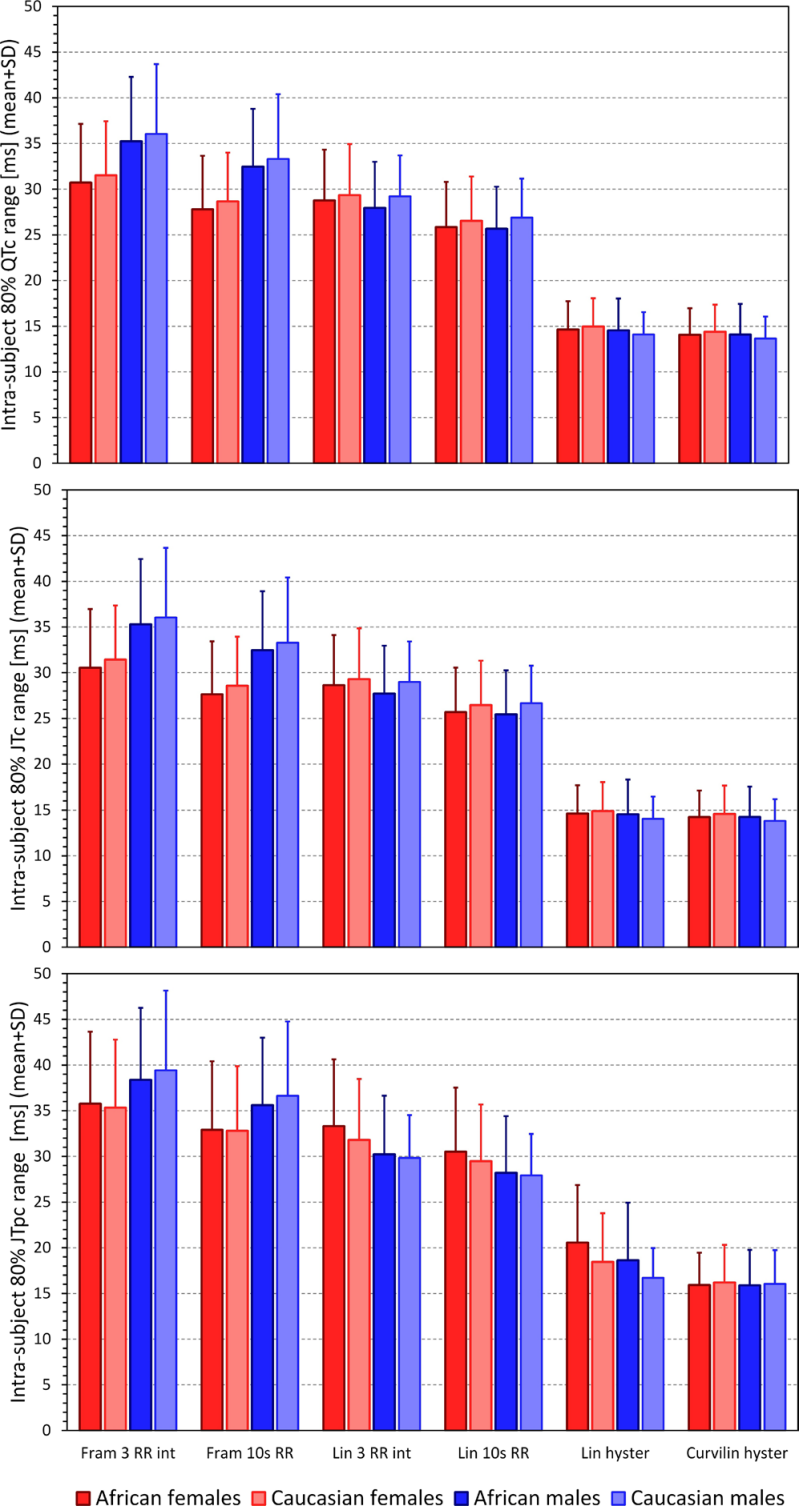
Summary of intra-subject 80^th^-percentile ranges of QTc (top panel), JTc (middle panel) and JTpc (bottom panel) intervals obtained with different rate correction formulas. The layout of the Figure and the meaning of the correction formula labels is the same as in Fig. 2. In each of the study sub-populations, the intra-subject 80^th^-percentile ranges of all the intervals obtained with curvilinear model + hysteresis correction were statistically significantly smaller compared to all other correction possibilities (p < 0.001 for all).

The gradual reduction in the intra-subject SD of JTpc intervals is shown in more detail in the scatter diagrams in Fig. 3. Figure 4 shows that the results of the reduction of the intra-subject SD of rate corrected intervals was replicated when studying the 80% ranges of the rate corrected intervals.

This analysis confirms that accuracy of the curvilinear correction models involving the rate hysteresis. Consequently, the results obtained with this correction model are used in the subsequent sections of the result presentation.

### Sex and rate differences in the QTc, JTc, and JTpc intervals

**Figure 5:**
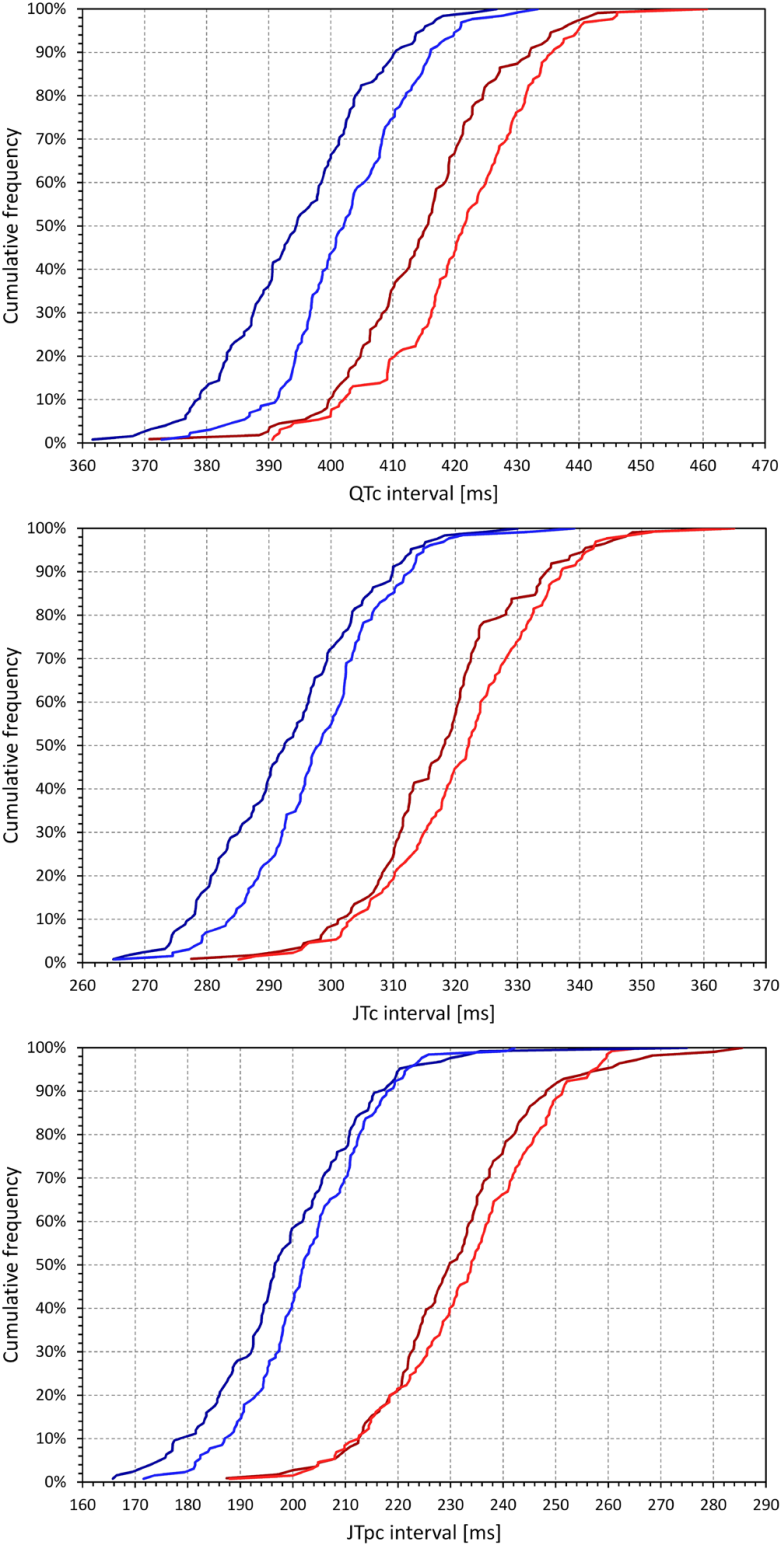
Cumulative distributions of QTc (top panel), JTc (middle panel), and JTpc (bottom panel) intervals in the investigated population. In each panel, the dark red, light red, dark blue, and light blue lines correspond to the measurements in African females, Caucasian females, African males, and Caucasian males, respectively. The data of subjects of other races are not shown.

Figure 5 shows the cumulative distributions of QTc, JTc and JTpc intervals distinguishing African and Caucasian females as well as African and Caucasian males. The corresponding numerical summaries of the measurements and the p-values of relevant statistical tests are shown in Table 1.

As expected, women had longer QTc interval than males. They also had longer JTc and JTpc intervals. All these differences were also highly statistically significant when testing the sex differences separately in subjects of African and Caucasian origin.

Compared to Caucasians, subjects of African origin also had shorter QTc, JTc, and JTpc intervals. However, the race difference was gradually diminishing. The largest difference was for QTc intervals where the race difference was highly significant (p < 0.001 for both sexes). The race difference of JTc interval was still statistically significant (p = 0.042 and p = 0.001 for females and males, respectively). The race difference of JTpc was only significant in males (p = 0.021).

**Figure 6:**
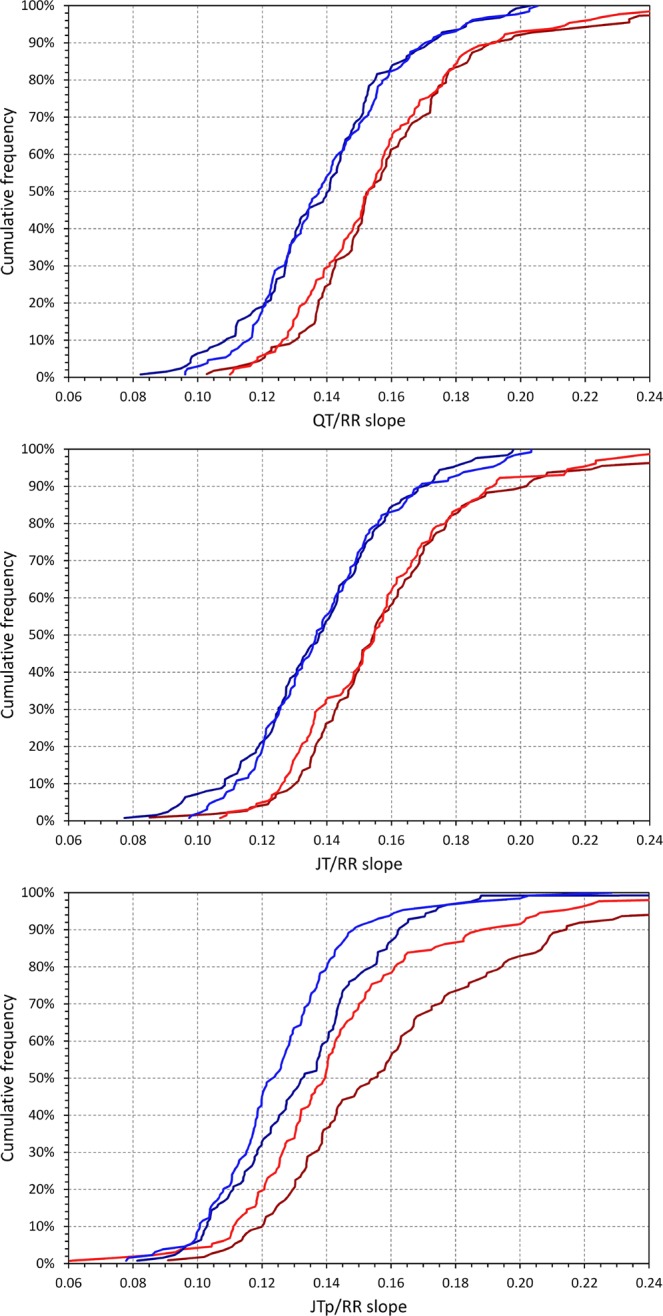
Cumulative distributions of QT/RR (top panel), JT/RR (middle panel), and JTp/RR (bottom panel) slopes of the curvilinear regression models in the investigated population. In each panel, the dark red, light red, dark blue, and light blue lines correspond to the measurements in African females, Caucasian females, African males, and Caucasian males, respectively. The data of subjects of other races are not shown.

**Figure 7:**
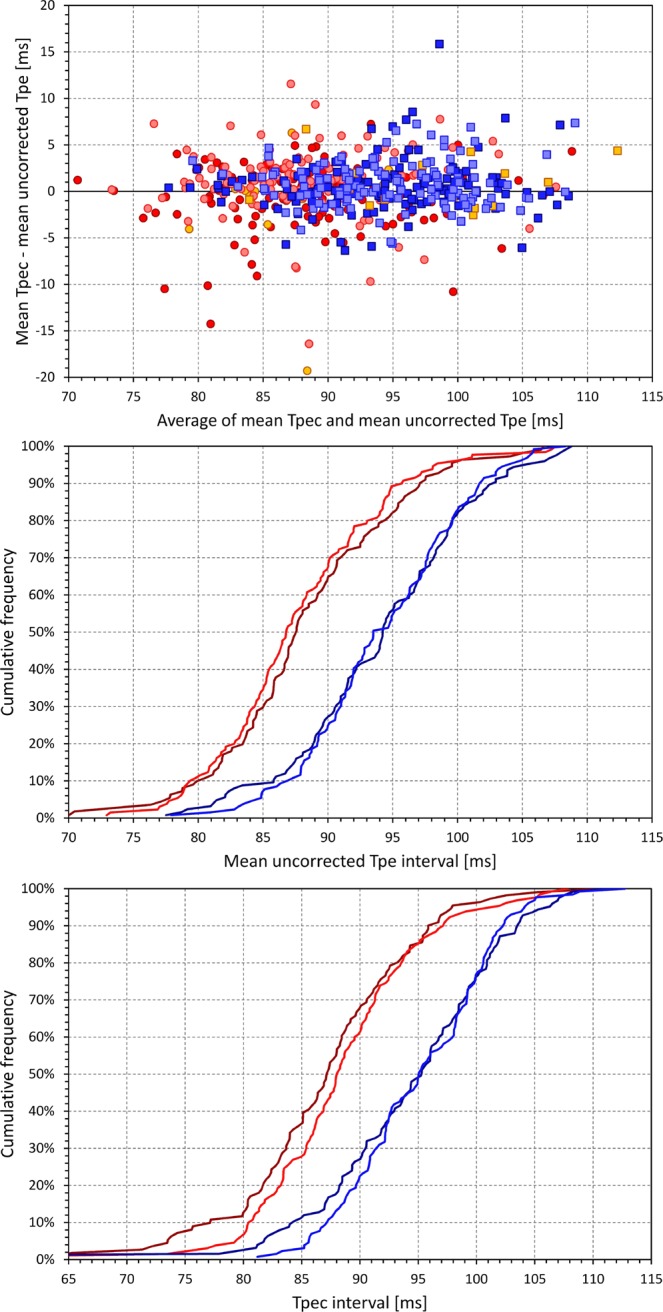
Top panel shows the Bland-Altman-like scatter diagram comparing intra-subject Tpec and uncorrected Tpe intervals. Their average is shown on the horizontal axis and their difference on the vertical axis. The dark red circles, light red circles, amber circles, dark blue squares, light blue squares, and amber squares correspond to the measurements in African females, Caucasian females, other race females, African males, Caucasian males, and other race males, respectively. The middle and bottom panels show cumulative distributions of Tpe (middle panel) and Tpec (bottom panel) intervals in the investigated population. In these panels, the dark red, light red, dark blue, and light blue lines correspond to the measurements in African females, Caucasian females, African males, and Caucasian males, respectively. The data of subjects of other races are not shown in the middle and bottom panels.

As shown in Table 1 and Fig. 6, women also had the QT/RR, JT/RR and JTp/RR relationships steeper than males (p < 0.001). The slope of the QT/RR and JT/RR relationship was not different between African and Caucasian subjects. However, the African subjects has steeper JTp/RR patterns compared to Caucasians (p = 0.02). This difference was more marked in women than men (see the bottom panel of Fig. 7).

### Tpeak-Tend interval and its sex difference

The Tpe interval was found practically heart rate independent. The top panel in Fig. 7 shows that there were only minimal differences between the Tpec interval and the uncorrected Tpe interval.

As it can be seen in the comparisons of the JT/RR and JTp/RR slopes, in approximately two thirds of the study population (171, 65% of females; 195, 72% of males; 140, 59% of African origin subjects; 209, 80% of Caucasians) the JT/RR slope was steeper than the JTp/RR slope. This means Tpe interval was increasing with increasing rate in some subjects but decreasing in others. In any case, however, these intra-subject relationships were shallow – on average, the difference between the JT/RR and JTp/RR slope within the same subject was only 2.2 ± 45.6% of the subject-specific JT/RR slopes.

The two bottom two panels of Fig. 7 show that the sex difference of the Tpe/Tpec interval was the exact opposite compared to QTc, JTc and JTpc intervals. Women were found to have the Tpe interval statistically shorter than men (p < 0.001) but there were no race differences in the Tpe duration.

### Dependency on age

**Figure 8:**
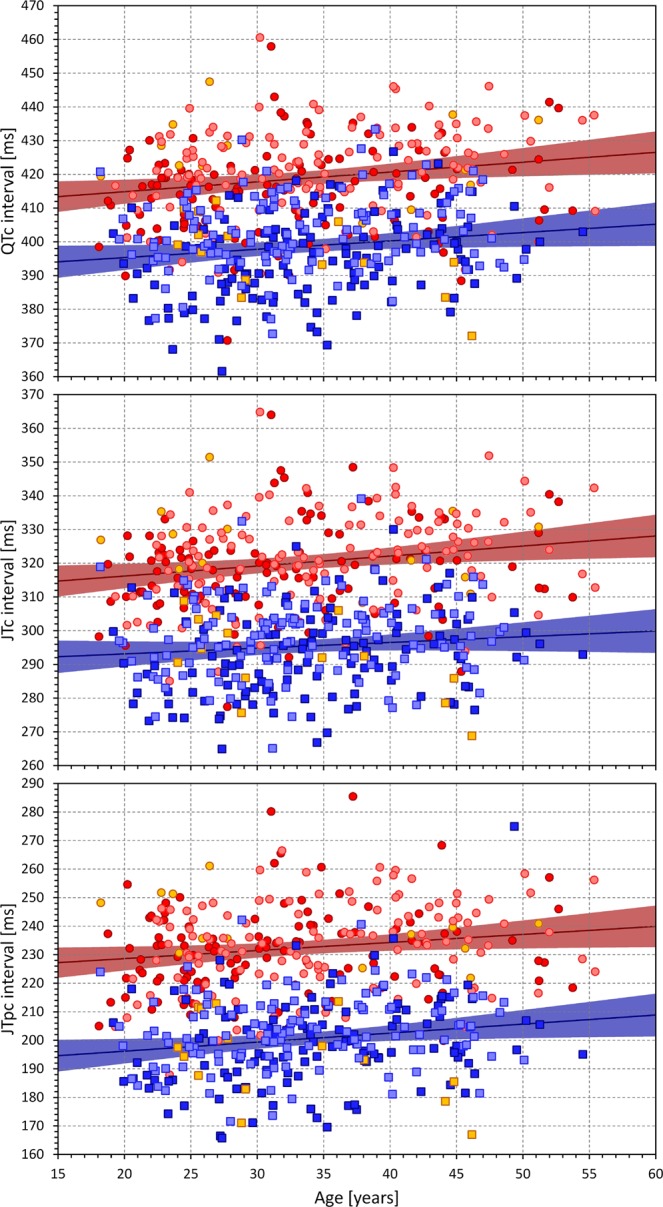
Age dependency of QTc (top panel), JTc (middle panel), and JTpc (bottom panel) intervals in the investigated population. In each panel, the dark red circles, light red circles, amber circles, dark blue squares, light blue squares, and amber squares correspond to the measurements in African females, Caucasian females, other race females, African males, Caucasian males, and other race males, respectively. In each panel, the solid red and solid blue lines show the linear regressions between the measured intervals and age in all females and all males, respectively. The red shaded and blue shaded areas are the 95% confidence intervals of the regression lines.

As seen in Fig. 8, QTc, JTc and JTpc intervals were mildly but significantly prolonging with advancing age. Overall, the strongest of these correlations was found for the JTpc intervals (p = 0.008 and p = 0.005 in females and males).

**Figure 9:**
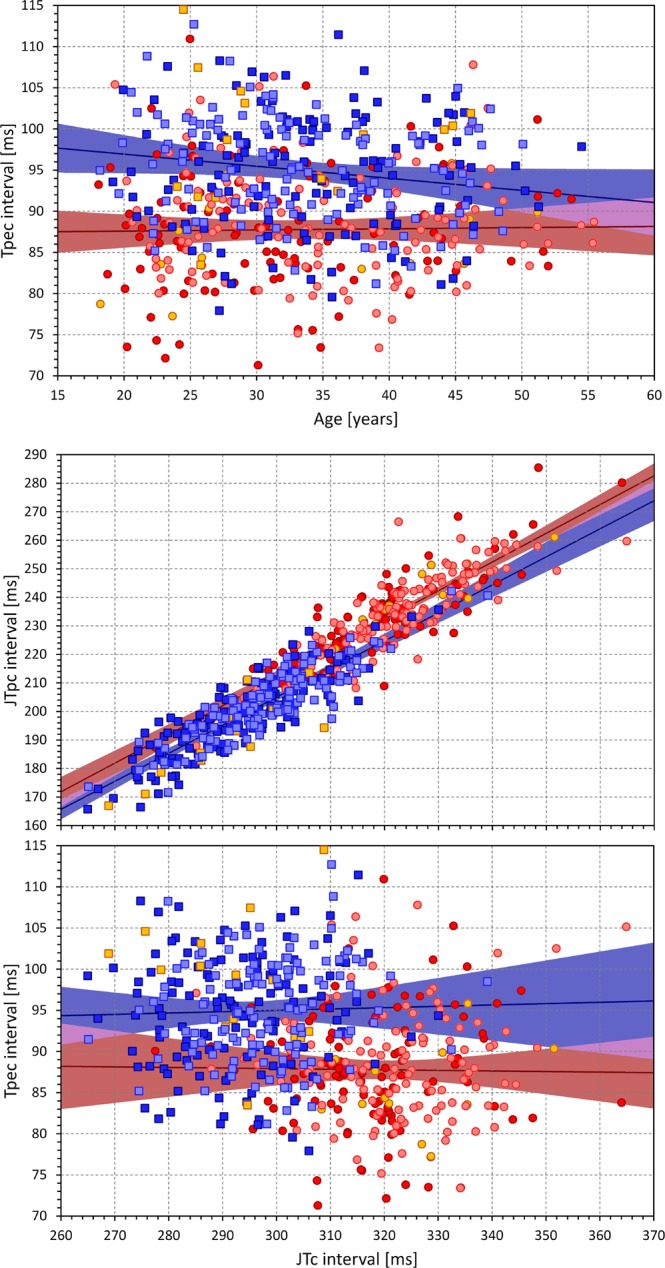
The top panel shows the age dependency of the Tpec intervals. The middle and bottom panels show the relationship between the JTpc (middle panel) and Tpec (bottom panel) and JTc intervals. The layout of the graphs and the meaning of the symbols are the same as in Fig. 8 (in the middle and bottom panel, linear regressions were calculated between the compared ECG intervals). The violet areas are the overlaps between the confidence intervals of the sex-specific regressions.

On the contrary, the Tpec interval was unrelated to age in females and was found decreasing with increasing age in males (top panel of Fig. 9). However, this was caused by a significant trend in Caucasian males (p = 0.015) while in African males, Tpec was not significantly related to age.

Additionally, the bottom panels of Fig. 9 shows that while JTc and JTpc intervals were closely correlated, Tpec intervals were relatively constant across the population (with the differences between sexes as already described) and statistically independent of JTc intervals. In the sub-populations of African females, Caucasian females, African males, and Caucasian males, the correlations between JTc and Tpec intervals were −0.062, −0.004, −0.023, and 0.121, respectively, none of which was even close to statistical significance.

### Rate hysteresis of QT, JT, and JTp intervals

We have not found any systematically significant correlations (i.e., correlations reproducible in different sub-groups) between age and QT/RR, JT/RR, and JTp/RR slopes.

On the contrary, we have found systematic difference between the hysteresis time constants of the JT/RR adaptation and JTp/RR adaptation. After a heart rate change, the time needed for the adaptation of the JTp interval was, on average, some 11% longer than the time needed for the JT adaptation (7%, p = 0.007; 16%, p < 0.001; 8%, p = 0.001; and 13%, p < 0.001; for African females, Caucasian females, African males, and Caucasian males, respectively).

**Figure 10:**
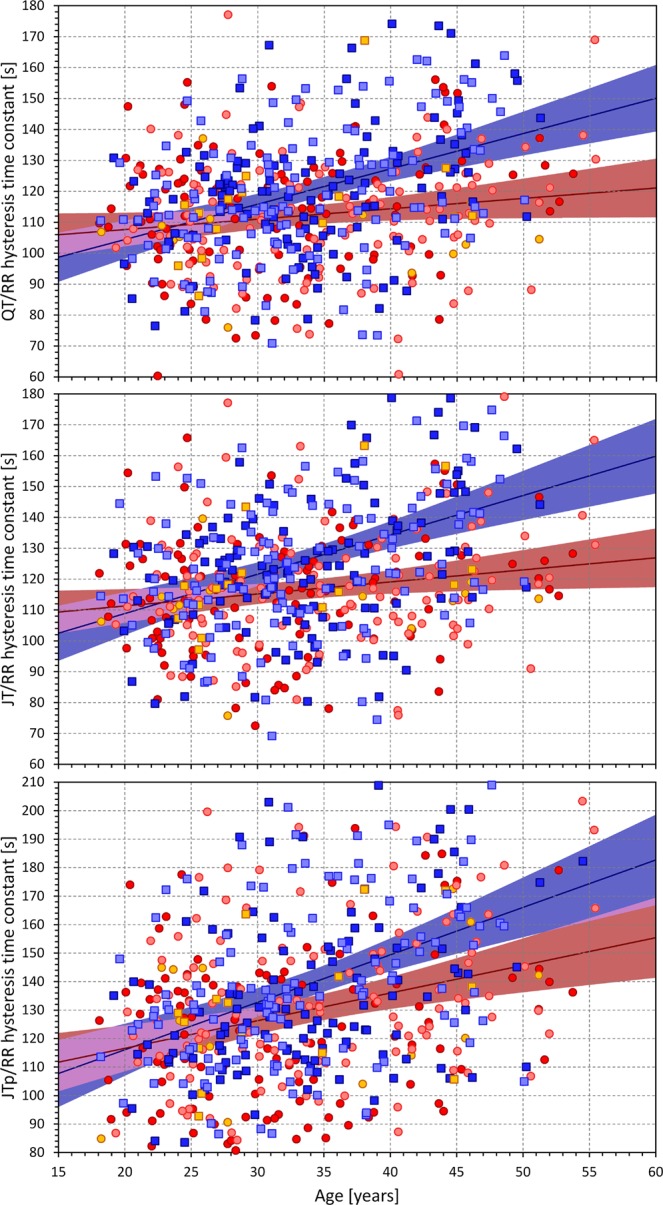
Age dependency of the QT/RR (top panel) JT/RR (middle panel) and JTp/RR (bottom panel) hysteresis time constants. The layout of the graphs and the meaning of the symbols are the same as in Fig. 8. The violet areas are the overlaps between the confidence intervals of the sex-specific regressions.

As seen in Fig. 10, we have also found statistically significant prolongation of the JT/RR and JTp/RR hysteresis time constants with advancing age (p between <0.001 and 0.05, and between <0.001 and 0.024 in the individual sex- and race-specific sub-groups for JT/RR hysteresis and JTp/RR hysteresis, respectively).

## Discussion

The study provides observations that appear important for future investigations of the clinical value of JTp and Tpe intervals.

Similar to the QTc interval^8^, JTc, JTpc and Tpec intervals are sex dependent. However, while JTpc intervals are longer in females than in males, the Tpec intervals are longer in males than in females. In future clinical studies of the QT interval sub-sections, sex of the subjects needs to be considered and appropriately statistically evaluated. For instance, an observation that longer Tpe interval is an indicator of increased arrhythmic risk might be biased if based on a general middle-aged population including both sexes. Since generally, arrhythmic risk is lower in middle-aged females compare to males, the shorter Tpe interval in females might lead to erroneous extrapolations. Clinical studies of QTc, JTc and JTpc intervals should also consider race differences. Similarly, sex and race should be considered in power sample calculations of future study designs^22,23^. The same considerations apply to the studies using drug-induced JTpc interval changes to differentiate between pure blockers of delayed potassium rectifier and drugs with multiple ion channel effects^4,5^. If such studies are based on parallel design, careful sex and race-correspondence between study arms is needed to avoid unfounded conclusions.

Our data also suggest that the Tpe interval is not systematically heart rate dependent. The controversy of previous studies which did and did not found Tpe heart rate dependency^1^ seems also explained by our data. If Tpe prolongs or shortens with increasing heart rate differently in different subjects, studies of smaller populations are bound to be inaccurate and substantially dependent on the composite of the investigated population. The subject-specific nature of the Tpe/RR relationship also influences the observations of the rate dependency if these are based on singular readings or only small number of ECGs in each individual, leading to the same problem as known with the diverse multitude of previously proposed QT correction formulae.

Future Tpe studies need to note that even if the Tpe/RR dependency is incorporated into the investigation, it is much shallower than that of the QT (or JT) intervals. Correcting Tpe for the underlying heart rate using Bazett or Fridericia formula^24^ is highly erroneous and makes the corrected values positively correlated with heart rates. Since increased heart rate is a known risk factor, highly biased data of risk prediction by Tpe might be generated by these correction formulae.

While the main thrust of the study was the investigation of the sex and race differences in the ECG intervals, the comparison of the rate correction methodology also has practical implications. In particular, regardless of whether we used a fixed correction (Framingham formula) or individually optimised linear correction with instantaneous heart rate measurements, the accuracy of the correction was much lower compared to the approaches that incorporated the rate hysteresis. This observation was independent of the sex- and race-defined sub-groups of the population. While this is well in agreement with previous observations^9,18–20^ correction for heart rate hysteresis is only rarely used in electrocardiographic investigations, probably because correcting for the rate hysteresis is beyond usual day-to-day possibilities of clinical practice. Nevertheless, attention needs to be given to the phenomenon and if hysteresis correction is not used, ECG measurements should be made after prolonged episodes of stable heart rate. This is not necessarily trivial to achieve. Although physical reasons for heart rate differences can be eliminated by maintaining undisturbed position for a sufficiently long period before ECG recording, psychological and mental reasons for heart rate fluctuations are beyond clinical control whilst their effects might be substantial^20^. Thus, stability of preceding heart rate needs to be checked, e.g. by obtaining multiple closely coupled ECG recordings. If neither hysteresis correction is used nor the stability of preceding heart rate assured, substantial imprecision of the QTc, JTc, or JTpc intervals might be created. Figure 4 shows that this imprecision may span some tens of milliseconds even when eliminating 20% of outlying values. This might have profound implications for power sample calculations of clinical studies^22,25^.

In addition to these implications for future investigations, we have also made physiologic observations. The gradual prolongation of QTc, JTc and JTpc intervals with advancing age corresponds to previous observations^26^ albeit our data suggest somewhat lesser degree of age influence. Previous publications might have been influenced by inaccuracies in rate corrections.

Not only heart rate dependency but also inter-subject differences of the JT (and QT) intervals are primarily driven by the rate dependency and individuality of the JTp intervals. Our observations suggest that the Tpe interval is a relatively constant extension of the JTp interval and that it little responds to physiologic regulation.

The somewhat prolonged heart rate hysteresis of the JTp interval compared to the JT interval seems to suggest that after heart rate change, an overall change in the action-potential durations of ventricular myocytes occurs faster than the equilibrium of repolarisation distribution across the ventricles. The strong dependency of the hysteresis time constants on age suggests a link to the autonomic system which is known to be less responsive with advancing age^27^. This also suggests that the time constant of the JTp rate hysteresis might offer a direct measure of autonomic influence at the ventricular level and that it should be investigated in future risk assessment studies^28^.

Although the data of healthy subjects cannot be interpreted in terms of myocardial abnormalities, the fact that Tpe interval is neither systematically responding to heart rate nor shows any age-related changes does not seem to be fully consistent with the suggestion that this interval represents repolarisation heterogeneity. Also, more direct measures of repolarisation heterogeneity, namely the T wave morphological disparities, have previously been reported increased in females compared to males^29^, again suggesting that the link between the Tpe interval and repolarisation spatial dispersion might have previously been overestimated.

### Limitations

Since the study investigated healthy subjects, we cannot comment on the extrapolation of the results to cardiac patients with repolarisation abnormalities. We thus cannot provide any clinical outcome data. Nevertheless, it seems evident that increased accuracy of ECG data, in terms of both sex- and race-differences and rate correction methodology, might only increase the power of clinical investigations. Indeed, the importance of QT/RR hysteresis was previously reported in an outcome study of cardiac patients^30^.

Previous publications used a variety of methods to determine the T wave peak. Of these, we selected only one method since it was used in important repolarisation studies^4,5^. It is possible that different methods would produce largely different results in diseased hearts although we believe that in healthy subjects, the method used in the study leads to the most physiologically relevant assessment that has recently been found to lead to the lowest variability of the rate corrected intervals^16^.

While we were able to determine the sex of the study subjects with certainty (the population did not contain any sex-transversal cases) the race was derived from self-declarations. We have no genetic information of the subjects to determine their race objectively. The initial pharmacology studies did not provide echocardiographic and other imaging data that would allow us to relate the ECG measurements to myocardial size measurements.

Exponential decay models have previously been used to study QT/RR hysteresis^18^. It is theoretically possible that other models would be more appropriate for studying the JTp/RR hysteresis. Nevertheless, the small regression residuals that we observed suggest that any improvement in the hysteresis assessment would only be very modest. In the evaluation of the heart rate correction possibilities, we have used the Framingham formula and individual-specific linear models rather than Fridericia formula and corresponding log-linear models. Since the uncorrected QT, JT, and JTp intervals differ substantially in their population mean values, corrections based on logarithmic transformation lead to substantial instabilities of correction coefficients^31^.

Since the source pharmacological studies were conducted in US clinical centres, subjects of African origin were African Americans. We have no data to compare our results to observations to native residents of African countries. Finally, the age span of the investigated population ranged only from 18 to 55 years. This potentially limits the analyses of age dependency.

## Conclusion

Despite these limitations, the data of this study permit us to conclude that QTc intervals and QT sub-sections exhibit clear sex and race differences. Surprisingly, while females have longer QTc, JTc and JTpc intervals, their Tpe intervals are shorter compared to those in males. The heart rate dependency of Tpe intervals is very shallow and different subjects show both positive and negative correlations of Tpe with the underlying rate. The speed of hysteresis adaptation of the JTp intervals to heart rate changes might provide direct probe into autonomic influence at the level of ventricular myocardium.

## References

[1] Malik M, Huikuri H, Lombardi F, Schmidt G, Zabel M (2018). Conundrum of the Tpeak-Tend interval. J. Cardiovasc. Electrophysiol..

[2] Tse G (2017). The Tpeak - Tend interval as an electrocardiographic risk marker of arrhythmic and mortality outcomes: A systematic review and meta-analysis. Heart Rhythm.

[3] O’Neal, W. T. *et al*. Association between QT-interval components and sudden cardiac death: The ARIC study (Atherosclerosis Risk in Communities). *Circ*. *Arrhythm*. *Electrophysiol*. **10**, 10.1161/circep.117.005485 (2017). 10.1161/CIRCEP.117.005485 PMC5659833 29030380

[4] Johannesen L (2014). Differentiating drug-induced multichannel block on the electrocardiogram: randomized study of dofetilide, quinidine, ranolazine, and verapamil. Clin. Pharmacol. Ther..

[5] Johannesen L (2016). Ability of late sodium or calcium current block to balance the ECG effects of potassium current block. Clin. Pharmacol. Ther..

[6] Seegers J, Hnatkova K, Friede T, Malik M, Zabel M (2017). T-wave loop area from a pre-implant 12-lead ECG is associated with appropriate ICD shocks. PLoS One.

[7] Hnatkova K (2018). Clinical value of different QRS-T angle expressions. Europace.

[8] Linde C (2018). Sex differences in cardiac arrhythmia: a consensus document of the European Heart Rhythm Association, endorsed by the Heart Rhythm Society and Asia Pacific Heart Rhythm Society. Europace.

[9] Hnatkova K (2019). Heart rate correction of the J-to-Tpeak interval. Sci. Rep..

[10] ICH Guideline (2001). Safety pharmacology studies for human pharmaceuticals S7A. Fed. Regist..

[11] Malik M (2012). Proarrhythmic safety of repeat doses of mirabegron in healthy subjects: a randomized, double-blind, placebo-, and active-controlled thorough QT study. Clin. Pharm. Therap..

[12] Hnatkova K (2009). Systematic comparisons of electrocardiographic morphology increase the precision of QT interval measurement. Pacing Clin. Electrophysiol..

[13] Johannesen L, Garnett C, Malik M (2014). Electrocardiographic data quality in thorough QT/QTc studies. Drug Saf..

[14] Guldenring D (2015). The derivation of the spatial QRS-T angle and the spatial ventricular gradient using the Mason-Likar 12-lead electrocardiogram. J. Electrocardiol..

[15] Johannesen L, Vicente J, Hosseini M, Strauss DG (2016). Automated algorithm for J-Tpeak and Tpeak-Tend assessment of drug-induced proarrhythmia risk. PLoS One.

[16] Hnatkova, K. *et al*. Detection of T wave peak for serial comparisons of JTp interval. *Front*. *Physiol*. **10**, 10.3389/fphys.2019.00934 (2019). 10.3389/fphys.2019.00934 PMC6670189 31402872

[17] Malik M, Hnatkova K, Kowalski D, Keirns JJ, van Gelderen EM (2013). QT/RR curvatures in healthy subjects: sex differences and covariates. Am. J. Physiol. Heart Circ. Physiol..

[18] Malik M, Hnatkova K, Novotny T, Schmidt G (2008). Subject-specific profiles of QT/RR hysteresis. Am. J. Physiol. Heart Circ. Physiol..

[19] Gravel Hugo, Jacquemet Vincent, Dahdah Nagib, Curnier Daniel (2017). Clinical applications of QT/RR hysteresis assessment: A systematic review. Annals of Noninvasive Electrocardiology.

[20] Malik M, Johannesen L, Hnatkova K, Stockbridge N (2016). Universal correction for QT/RR hysteresis. Drug Saf..

[21] Sagie A, Larson MG, Goldberg RJ, Bengtson JR, Levy D (1992). An improved method for adjusting the QT interval for heart rate (the Framingham Heart Study). Am. J. Cardiol..

[22] Malik M (2004). Sample size, power calculations, and their implications for the cost of thorough studies of drug induced QT interval prolongation. Pacing Clin. Electrophysiol..

[23] Zhang J, Machado SG (2008). Statistical issues including design and sample size calculation in thorough QT/QTc studies. J. Biopharm. Stat..

[24] Chua KC (2016). Tpeak-to-Tend interval corrected for heart rate: a more precise measure of increased sudden death risk?. Heart Rhythm.

[25] Garnett CE (2012). Methodologies to characterize the QT/corrected QT interval in the presence of drug-induced heart rate changes or other autonomic effects. Am. Heart J..

[26] Rautaharju PM, Mason JW, Akiyama T (2014). New age- and sex-specific criteria for QT prolongation based on rate correction formulas that minimize bias at the upper normal limits. Int. J. Cardiol..

[27] Malik M (2019). CrossTalk proposal: Heart rate variability is a valid measure of cardiac autonomic responsiveness. J. Physiol..

[28] Steger, A. *et al*. Polyscore of non-invasive cardiac risk factors. *Front*. *Physiol*. **10**, 10.3389/fphys.2019.00049 (2019). 10.3389/fphys.2019.00049 PMC6369149 30778303

[29] Smetana P, Batchvarov VN, Hnatkova K, Camm AJ, Malik M (2002). Sex differences in repolarization homogeneity and its circadian pattern. Am. J. Physiol. Heart Circ. Physiol..

[30] Pueyo E (2004). Characterization of QT interval adaptation to RR interval changes and its use as a risk-stratifier of arrhythmic mortality in amiodarone-treated survivors of acute myocardial infarction. IEEE Trans. Biomed. Eng..

[31] Hnatkova K, Johannesen L, Vicente J, Malik M (2017). Heart rate dependency of JT interval sections. J. Electrocardiol..

